# Symptomatic Ventricular Bigeminy and Trigeminy Associated With COVID-19

**DOI:** 10.7759/cureus.38650

**Published:** 2023-05-06

**Authors:** Janie Hu, Alexander T Phan, Buzand Oganesian, Hyungjin B Kim

**Affiliations:** 1 Internal Medicine, Arrowhead Regional Medical Center, Colton, USA; 2 Cardiology, Loma Linda University Medical Center, Loma Linda, USA

**Keywords:** internal medicine, cardiology, covid-19, trigeminy, bigeminy

## Abstract

Cardiac manifestations of COVID-19 are well-described in the current literature, although electrocardiogram analyses of COVID-19 patients are limited. The most common arrhythmias experienced by patients with COVID-19 include sinus tachycardia and atrial fibrillation. Ventricular bigeminy associated with COVID-19 is exceedingly rare and requires further studies to determine its incidence and clinical significance. Here, we present the case of a 57-year-old male with no prior cardiac history who was found to have COVID-19 and new-onset, symptomatic premature ventricular contraction bigeminy. This case highlights a rare potential association between COVID-19 and ventricular bigeminy/trigeminy.

## Introduction

The Coronavirus Disease 2019 (COVID-19) is caused by the severe acute respiratory syndrome coronavirus 2 (SARS-CoV-2) and has affected millions worldwide. It was declared a global pandemic in March 2020 by the World Health Organization [1-3]. COVID-19 primarily manifests in the lungs; however, extrapulmonary manifestations, including cardiac, have also been cited in the literature [1-3]. Some common cardiac manifestations of COVID-19 include myocardial injury, myocarditis, acute heart failure, acute coronary syndrome, and cardiac arrhythmias [1,3]. It has been well-established that the severity of COVID-19 directly correlates with an increased risk for cardiac manifestations [2]. Given the fact that COVID-19 has many cardiac-related manifestations, its further analysis is warranted.

The current literature is limited in terms of electrocardiogram (ECG) abnormalities in COVID-19 patients, but some studies have cited atrial fibrillation and sinus tachycardia as the most common arrhythmias associated with this disease entity [1,3]. Currently, there are a limited number of case studies that have cited ventricular bigeminy or trigeminy associated with COVID-19 [1-5]. Here, we present the case of a 57-year-old SARS-CoV-2-positive male with no prior cardiac history who presented to the emergency department with a headache, rhinorrhea, muscle spasms of the mid-to-upper back, and chest palpitations. A 12-lead ECG demonstrated ventricular bigeminy and trigeminy, while previous ECGs in the previous 2 months demonstrated sinus bradycardia. We aim to contribute to the current limited body of literature citing ventricular bigeminy as a cardiac manifestation of COVID-19.

## Case presentation

A 57-year-old-male with a past medical history of recent brainstem stroke 3 months prior, hypertension, basal cell carcinoma, gastroesophageal reflux disorder, bilateral glaucoma, colonic adenomas, and testicular hydrocele status post hydrocelectomy presented with complaints of a sudden onset constant, dull, occipital headache, rhinorrhea, mid-to-upper back pain, and chest palpitations that started the previous evening. The patient reported symptoms of “heart racing,” but when he checked his smart watch, his heart rate was read as 45 beats per minute. The patient also noted a pre-syncopal episode this morning prior to arrival at the emergency department, stating that he felt light-headed while seated in a chair. He reported that exertion worsened his palpitations. He denied nausea, vomiting, diaphoresis, dizziness, lightheadedness, cough, or shortness of breath. He also denied any history of anxiety disorders, thyroid disorders, alcohol use, caffeine or stimulant drug use; however, he admitted to a history of daily marijuana use. His home medications included oral aspirin, atorvastatin, omeprazole, hydrochlorothiazide, and meloxicam.

**Figure 1:**
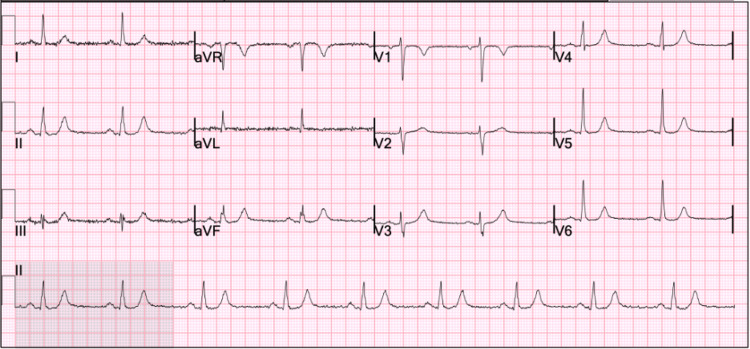
12-lead electrocardiogram from 2 months prior to admission demonstrating sinus bradycardia with a ventricular rate of 54.

**Figure 2:**
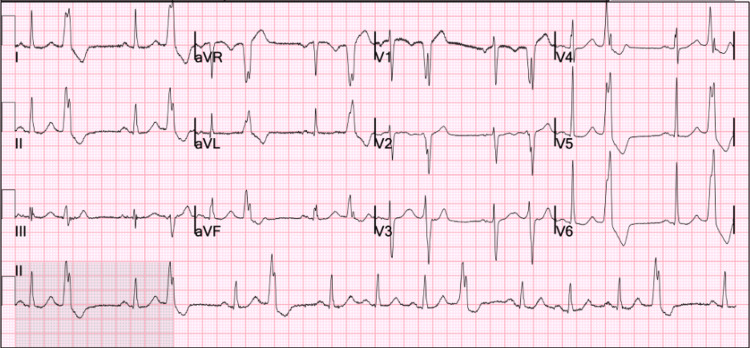
12-lead electrocardiogram on admission demonstrating ventricular bigeminy and electrocardiogram criteria for left ventricular hypertrophy and right atrial enlargement.

**Figure 3:**
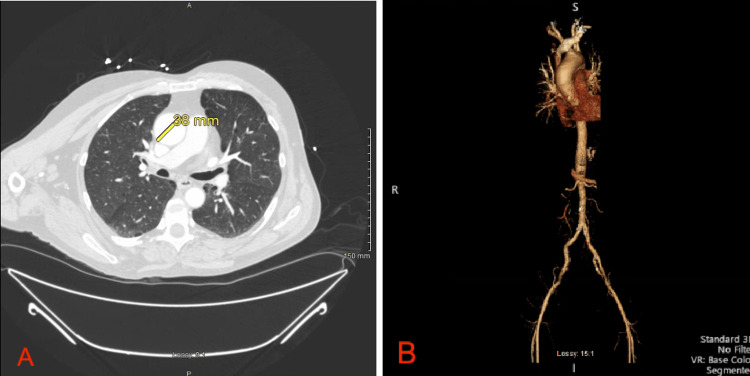
A: Axial section of a computed tomography angiogram of the chest demonstrating emphysematous lung parenchymal changes and an ectatic ascending aorta measuring 3.8 cm. B: Three-dimensional reconstruction of the chest, abdomen, and pelvic arterial vasculature. cm: centimeter; mm: millimeter

His initial vital signs included a blood pressure of 123/76 mmHg, pulse rate of 90, respiratory rate of 20, temperature of 99 °F, and oxygen saturation of 95% on ambient air. Initial laboratory studies demonstrated a positive SARS-CoV-2 polymerase chain reaction (GeneXpert) test; all other metabolic and blood counts were within normal limits. A urine toxicology screen was positive tetrahydrocannabinol. His previous 12-lead ECG from 2 months prior was reviewed, demonstrating sinus bradycardia with a ventricular rate of 54 beats per minute (Figure 1), and another ECG was obtained on admission and demonstrated ventricular bigeminy (Figure 2). A computed tomography angiogram of the chest, abdomen, and pelvis was obtained due to concern for aortic dissection and demonstrated an ectatic ascending aorta measuring 3.8 cm, emphysematous changes, fatty liver, and a hiatal hernia (Figure 3A-3B). A subsequent review of the patient’s transthoracic echocardiogram from his previous admission 2 months prior was significant for mild left ventricular hypertrophy and a mild ascending aorta dilatation of 4.1 cm.

**Figure 4:**
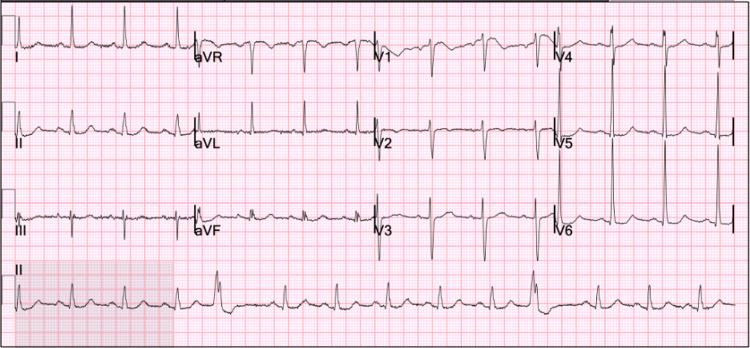
12-lead electrocardiogram prior to discharge from the emergency department demonstrating two premature ventricular complexes and electrocardiogram criteria for left ventricular hypertrophy and right atrial enlargement.

The patient was given oral acetaminophen 1000 mg once, which improved his headache and back pain. He was placed on telemetry monitoring, and the rhythm strip continuously demonstrated ventricular bigeminy and trigeminy for approximately four hours. Regarding his SARS-CoV-2 infection, as he was not requiring oxygen supplementation, he was started on a 5-day oral combination regimen of nirmatrelvir 300 mg and ritonavir 100 mg twice daily. Additionally, the patient had serum troponin-I levels measured every 4 hours for two occurrences, and both results were within laboratory reference ranges. A repeat ECG was obtained 5 hours after the initial presentation, showing two premature ventricular complexes in a 10-second period (Figure 4). At this point in time, the patient began to feel better, so he was discharged home with a plan for close follow-up with his primary care provider and a cardiologist.

## Discussion

Premature ventricular contractions (PVCs) occur when a heartbeat is initiated by the Purkinje fibers instead of the SA node. Some explanations include ectopic pacemaker cells, re-entrant signaling, or spontaneous depolarizations [6]. If a PVC alternates with a single sinus beat, it is referred to as bigeminy; if a PVC occurs after two sinus beats, it is referred to as trigeminy. In the majority of cases, PVCs are benign; however, at times, they can signify underlying pathology. Known etiologies of PVCs include excess catecholamines, stimulant drugs, structural and ischemic heart disease, and metabolic abnormalities including hypokalemia, hypomagnesemia, anemia, and hyperthyroidism [6]. A minority of patients experience symptoms, such as lightheadedness and palpitations in our patient's case. In clinically symptomatic cases, workup may be warranted to rule out serious cardiac pathologies, such as myocardial infarction or cardiomyopathy, and non-cardiac etiologies like those mentioned previously. A higher-risk population that may prompt investigation includes patients of advanced age, male sex, or African American descent, and patients with underlying hypertension, ischemic heart disease, or bundle branch block [6]. 

Symptomatic ventricular bigeminy and trigeminy have seldom been reported in the literature in association with COVID-19. Despite the dearth of research, we hypothesize that the mechanism of bigeminy and trigeminy in COVID-19 is related to increased catecholamines in the setting of an inflammatory state from infection, as it is well known that COVID-19 is often related to increased production of inflammatory cytokines, sometimes even leading to cytokine storm. Physicians should consider COVID-19 as a rare potential etiology of symptomatic PVCs noted on electrocardiograms. However, due to the unknown incidence and clinical significance of this pathology, symptomatic PVCs secondary to COVID-19 should be treated as a diagnosis of exclusion.

A detailed history should be obtained, including symptoms associated with palpitations, medical history, medications, and drugs (especially with stimulant properties). Subsequently, a physical exam should be performed and an electrocardiogram and labs should be obtained. The electrocardiogram may reveal PVCs but importantly may show alternate pathologies such as acute or old ischemic changes, electrolyte abnormalities (e.g., peaked T waves in hyperkalemia), or structural abnormalities (eg, left axis deviation in left ventricular hypertrophy). Important labs to draw include a complete blood count to evaluate for anemia, a basic metabolic panel to identify electrolyte disturbances, and thyroid-stimulating hormone to screen for thyroid disorders. If a patient has new-onset symptomatic PVCs in the setting of COVID-19 infection and workup is grossly negative for alternative causes, this may suggest that COVID-19 is the etiology of the arrhythmia - this was the case for our patient. And therefore, the PVCs may be self-limited to the COVID-19 infection. Patients should be closely followed up in the primary care setting until resolution.

## Conclusions

Symptomatic ventricular bigeminy and trigeminy have seldom been reported in the literature in association with COVID-19. The mechanism for this may be related to excess catecholamine and cytokine production. Physicians should consider COVID-19 as a rare potential etiology of premature ventricular complexes in the setting of an otherwise negative workup. Future studies may aim to study etiopathology and risk factors for the development of symptomatic ventricular bigeminy, trigeminy, and quadrigeminy in a larger cohort of COVID-19 patients.
